# Metabolic Research in Aquatic Animal Nutrition, Physiology and Disease

**DOI:** 10.3390/metabo14010022

**Published:** 2023-12-28

**Authors:** Yan He, Qingsong Tan, Qingchao Wang

**Affiliations:** 1 College of Fisheries, Huazhong Agricultural University, Wuhan 430070, China; heyan@mail.hzau.edu.cn (Y.H.); tanqs2000@mail.hzau.edu.cn (Q.T.); 2 Engineering Research Center of Green Development for Conventional Aquatic Biological Industry in the Yangtze River Economic Belt, Ministry of Education, Wuhan 430070, China; 3 Key Lab of Freshwater Animal Breeding, Ministry of Agriculture and Rural Affair/Key Laboratory of Agricultural Animal Genetics, Breeding and Reproduction, Ministry of Education, Wuhan 430070, China

Aquaculture provides a significant amount of high-quality protein for human consumption and is one of the most efficient protein production industries. The nutritional metabolism of aquatic animals plays a crucial role in the flourishing development of aquaculture. Early studies primarily focused on the basic nutritional requirements of aquatic animals, such as protein, fat, and carbohydrates. Protein was determined to be the most important component in aquafeed, having profound effects on the growth and health of aquatic animals. However, in recent years, a shortage of traditional protein sources such as fish meal and soybean meal has hindered the global development of the aquafeed industry [1]. Moreover, the metabolism of aquatic animal proteins, carbohydrates, and lipids is closely linked to their physiological changes and disease occurrence. This Special Issue, “Metabolic Research in Aquatic Animal Nutrition, Physiology, and Disease”, includes 15 original research papers from different countries. These papers investigate the effects of alternative protein sources in feed on the metabolism of aquatic animal organisms, the regulation of nutrition metabolism in aquatic animals by feed additives, the metabolic response mechanisms of aquatic animal diseases, and the influence of environmental pressures on the nutritional metabolism of aquatic animals.

Compared to protein sources from terrestrial animals and plants, fish meal is the most important protein source for aquaculture due to its high-quality protein, balanced amino acid profile, and low levels of anti-nutritional factors [2]. However, the rapid development of the aquaculture industry has stimulated a significant demand for fish meal, leading to a continuous shortage in its supply. In fact, from 1950 to 2010, approximately 27% (around 20 million tons per year) of the fish caught by global marine fisheries were utilized to produce products such as fishmeal and fish oil. Notably, 90% of these fish were of food grade or premium quality. This indicates a certain inadequacy in our utilization of fish to sustain ourselves, necessitating a more rational use of fishery resources [3], Therefore, the exploration of alternative protein sources to replace fish meal has become a research hotspot in recent years [4]. Various cottonseed protein products have been investigated as a potential substitute for fish meal in the diets of various fish species [5,6,7]. Zhang et al. found that enzymatically hydrolyzed cottonseed protein concentrate (ECP), as a cost-effective and easily obtainable plant protein, could be used as a substitute for 15.56% of fish meal without compromising the growth of largemouth bass (*Micropterus salmoides*), and it improved their antioxidant capacity and immune status (contribution 1). Similarly, replacing FM with cottonseed meal protein hydrolysate had similar effects on blunt-snout bream (*Megalobrama amblycephala*) [8]. Cottonseed meal (CSM) has also been successfully used as a replacement for 20% of fish meal without affecting the muscle nutritional deposition in golden pompano (*Trachinotus ovatus*) (contribution 2). Furthermore, Liu et al. incorporated cottonseed protein concentrate (CPC) as a sole protein source in grass carp (*Ctenopharyngodon idellus*) feed and evaluated its nutritional value and optimal protein level. Their results showed that a protein level of 37.69% in the CPC4 group enhanced the growth performance and health status of grass carp, while high protein levels (CPC5 and CPC6) resulted in relatively poor growth performance (contribution 3). This could be attributed to a decrease in the feeding rate and increased energy expenditure on deamination of excess protein, which increased the nitrogen metabolism burden and negatively impacted fish growth [9]. Wheat gluten can safely replace 100% of fish meal and 61.2% of soy protein concentrate in the diet of red spotted grouper (*Epinephelus akaara*) (contribution 4). Similar findings were also documented in a prior investigation, wherein wheat gluten was partially substituted for dietary fish meal in large yellow croaker [10]. Enzymatic chicken plasma (ECP) derived from enzymatic hydrolysis of poultry by-products is a high-quality animal protein source for most aquatic organisms. Its rich content of amino acids such as arginine, alanine, and taurine can improve the feed acceptance of crustaceans; as such, it is considered a suitable substitute for fish meal [11]. Hlordzi et al. investigated the effects of ECP on the growth performance, digestive enzyme activity, and gene expression of Pacific white shrimp (*Litopenaeus vannamei*), finding that a dietary inclusion level of 32.4% crude protein in ECP1 was more suitable for the growth of juvenile shrimp (contribution 5). Furthermore, Yang et al. explored the utilization of *Clostridium autoethanogenum* protein (CAP), produced through bacterial metabolism of steel mill off-gas, as a replacement for fish meal. CAP successfully replaced 67.1-68.0% of the fish meal in the feed (contribution 6), though the incorporation of CAP into diets necessitates the supplementation of exogenous amino acids, particularly arginine, to maintain an optimal amino acid composition [12,13,14,15]. In addition to providing a range of feasible options for substituting protein sources in the aquaculture industry, aiming to improve feed utilization efficiency and reduce production costs, these studies have positive implications for environmental protection and waste utilization.

Another effective way to conserve protein is to fully utilize the energy effects of lipids and carbohydrates in feed [16,17]. However, higher levels of fat or glucose in the feed can lead to abnormal fat deposition in the liver, directly impacting the health of fish as well as their yield and economic benefits [18,19,20]. Deng et al. found that adding phytosterol glycosides to high-fat feed promoted fat decomposition in the liver of hybrid groupers (♀*Epinephelus fuscoguttatus* × ♂*Epinephelus lanceolatu*), activated the glycolytic pathway, enhanced the utilization of feed energy by fish, and supported the non-specific immune defense mechanisms in fish (contribution 7). Xia et al. demonstrated that regulating endoplasmic reticulum stress in spotted sea bass using 4-PBA, an endoplasmic reticulum stress inhibitor, reduced fat accumulation caused by high-fat feed (contribution 8). Similar results were also reported in some previous studies [21,22]. By combining liver proteomics and lipidomics, Xue et al. investigated the key signaling pathways and biomarkers associated with metabolic fatty liver disease in largemouth bass. They identified FABP1, ABCA1, and VDAC1 as potential biomarkers for the treatment of metabolic fatty liver disease in largemouth bass (contribution 9).

Certain feed additives also have significant benefits for the metabolic health of fish [23,24,25]. Wu et al. investigated the effects of soybean lecithin (SBL) on the growth, blood parameters, immune capacity, antioxidant capacity, and intestinal barrier function of juvenile largemouth bass. They found that 4% SBL supplementation improved these indicators (contribution 10). Song et al. examined the impact of dietary lactoferrin (LF) supplementation on the growth performance and intestinal health of juvenile orange-spotted grouper (*Epinephelus coioides*) fed a high-soybean meal (SBM) diet. The addition of appropriate LF improved the growth rate of fish, reduced the content of harmful substances in the intestine, and increased the diversity of beneficial bacteria (contribution 11). Similarly, research comparing the intestinal microbiota of grass carp fed different diets showed that diet can modulate the stability of the intestinal microbiota by altering its composition and interspecies interactions, thus regulating the growth metabolism of fish (contribution 12). Broughton et al. focused on the development and addition of a novel oil extracted from genetically modified (GM) oilseeds into Atlantic salmon (*Salmo salar*) feed. Phospholipid profiling analysis revealed that the long-chain polyunsaturated fatty acids (LC-PUFAs), EPA, and DHA derived from GM oilseeds could alter the lipid structure within fish, providing a theoretical basis for supplementing and replacing current terrestrial oilseeds and fish oil (contribution 13).

The water temperature and light intensity are also crucial to the growth and metabolism of fish. Chen et al. have proved that chronic low temperature affects the glucose and lipid metabolism in the liver of freshwater drum (*Aplodinotus grunniens*), with the miR-1/AMPK signaling pathway playing an important role in regulating these processes, providing a theoretical basis for freshwater drum’s resistance to low temperature (contribution 14). However, time-restricted feeding (TRF), which has remarkable effects on metabolic regulation caused by circadian rhythm disruption in mammals, failed to mitigate the negative effects of continuous light exposure induced by artificial night light on rainbow trout fry, resulting in increased body fat accumulation and significantly decreased muscle LC-PUFA ratio (contribution 15).

In conclusion, the research papers in the Special Issue “Metabolic Research in Aquatic Animal Nutrition, Physiology and Disease” provide valuable theoretical foundations for protein substitution in the field of aquatic animal nutrition and metabolism. They also delve into the occurrence of metabolic diseases and immune mechanisms in aquatic animals. This in-depth exploration is of great significance in the development of functional feed ingredients or efficient feeds, and in driving the sustainable prosperity of the aquaculture industry.
